# Fourier Transform Mass Spectrometry and Nuclear Magnetic Resonance Analysis for the Rapid and Accurate Characterization of Hexacosanoylceramide

**DOI:** 10.3390/ijms17071024

**Published:** 2016-06-28

**Authors:** Charles W. Ross, William J. Simonsick, Michael J. Bogusky, Recep W. Celikay, James P. Guare, Randall C. Newton

**Affiliations:** 1 Merck & Co. Inc., Merck Research Laboratories, Dept. of Medicinal Chemistry, West Point, PA 19486, USA; mjbogusky@gmail.com (M.J.B.); jpguare@gmail.com (J.P.G.); 2 DuPont Marshall R & D Laboratories, Philadelphia, PA 19146, USA; William.Simonsick@lubrizol.com (W.J.S.); recep.w.celikay@dupont.com (R.W.C.)

**Keywords:** ceramides, sphingolipids, adjuvants, Fourier transform ion cyclotron resonance mass spectrometry (FTICR MS), nuclear magnetic resonance (NMR), direct laser desorption ionization, electrospray ionization

## Abstract

Ceramides are a central unit of all sphingolipids which have been identified as sites of biological recognition on cellular membranes mediating cell growth and differentiation. Several glycosphingolipids have been isolated, displaying immunomodulatory and anti-tumor activities. These molecules have generated considerable interest as potential vaccine adjuvants in humans. Accurate analyses of these and related sphingosine analogues are important for the characterization of structure, biological function, and metabolism. We report the complementary use of direct laser desorption ionization (DLDI), sheath flow electrospray ionization (ESI) Fourier transform ion cyclotron resonance mass spectrometry (FTICR MS) and high-field nuclear magnetic resonance (NMR) analysis for the rapid, accurate identification of hexacosanoylceramide and starting materials. DLDI does not require stringent sample preparation and yields representative ions. Sheath-flow ESI yields ions of the product and byproducts and was significantly better than monospray ESI due to improved compound solubility. Negative ion sheath flow ESI provided data of starting materials and products all in one acquisition as hexacosanoic acid does not ionize efficiently when ceramides are present. NMR provided characterization of these lipid molecules complementing the results obtained from MS analyses. NMR data was able to differentiate straight chain versus branched chain alkyl groups not easily obtained from mass spectrometry.

## 1. Introduction

Ceramide is a central unit of all sphingolipids, a large class of membrane lipids that function as recognition sites on cell membrane surfaces and constitute a major component of nerve tissue. Ceramides formed by the metabolism of sphingolipids may function as signal transducers to mediate a variety of cellular processes. Previous studies suggest that ceramide is a key signaling molecule generated in response to stresses that mediate growth arrest, differentiation, apoptosis or an immune response [1,2,3,4]. Several glycosphingolipids have been identified that display immunomodulatory [5,6,7] and anti-tumor activities [8,9]. Considerable interest has been generated in the use of these molecules as potential adjuvants designed to enhance the immune response in humans when administered with vaccine preparations. Much effort is being devoted to the development of both safe and clinically-effective adjuvants for use in humans. A variety of analytical and enzymological techniques have been used to identify, characterize, and quantify these compounds [10].

Lipid and ceramide analyses have been performed by various techniques such as: derivatization with an ultraviolet chromophore or fluorescence tag for subsequent high performance liquid chromatography (HPLC) separation and UV analysis [11,12,13,14,15,16], HPLC separation with evaporative light scattering detection [17,18,19,20], fast atom bombardment ionization [21,22], and electrospray ionization mass spectrometry [23,24,25,26,27,28,29,30]. Derivatization of the hydroxyl or amine groups with a chromophore suitable for UV detection provides reliable quantitative results but is time consuming. The derivatized samples are typically unstable and must be analyzed rapidly before decomposition. Evaporative light scattering detection with HPLC separation provides direct analysis without derivatization and is ideal for compounds that lack a chromophore. Detection is a function of the amount of light scattered from the molecule and relates to the size or mass of the molecule. Quantitation is only possible using standards since a direct relationship to mass is not possible. Thin layer chromatography (TLC) is more commonly used for the separation and identification of ceramides [31]. Spot identification is usually done by staining and quantitation by densitometry. The capacity of TLC is low compared to HPLC for sufficient separation. Identification in all of these cases is determined by time or place of elution. Mass spectrometric detection has shown the most promise in identifying ceramides by mass measurement and product ion analysis from collisionally activated dissociation [21,22,29,30]. Normal phase HPLC coupled to atmospheric pressure chemical ionization (APCI) proved useful in the measurement of ceramides owing to the lack of salt adduction and ion suppression with APCI. However, APCI can induce fragmentation and one must be cautious of applying high voltages to the LC eluent flow of isooctane and ethyl acetate used to solubilize these samples [32]. Recently, an optimized coaxial electrospray ionization method [33], originally applied to polymer analysis [34], demonstrated superior results over conventional mass spectrometry methods for phytosphingosine, hexacosanoic acid and their reaction products. In all cases, we found that sample preparation is critical to obtaining reasonable results.

Herein, we discuss the rapid analysis of phytosphingosine and its products from reaction with hexacosanoic acid. Direct laser desorption ionization and sheath flow electrospray ionization do not require stringent sample preparation. These methods are compared to standard single needle electrospray ionization. High field NMR analyses are used for structural confirmation and to determine approximate relative ratios of starting material to desired product. High resolution ^13^C NMR analysis is used to identify straight chain or branched chain alkyl groups, which is not easily determined by MS or MS/MS experiments. Using these sophisticated analytical techniques, all the starting materials and reaction products can be identified and quantified.

## 2. Results and Discussion

### 2.1. Analysis of Starting Materials

**Figure 1:**
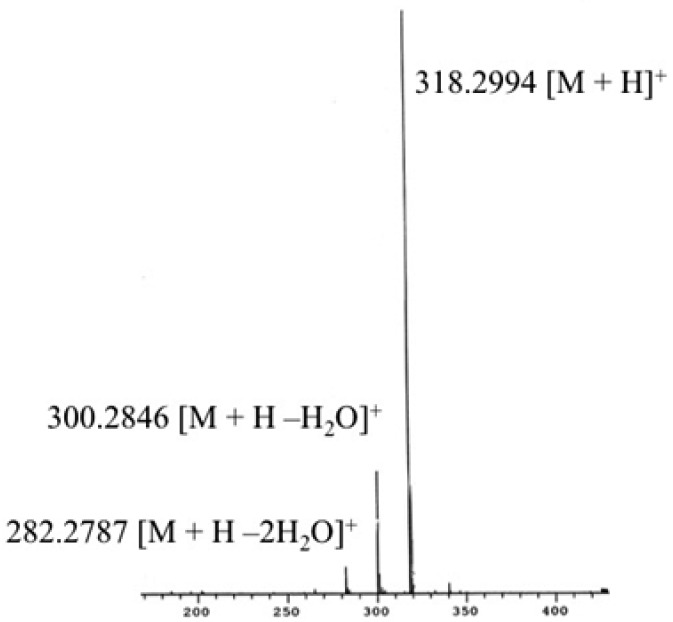
Standard monoelectrospray ionization mass spectrum of phytosphingosine. Increased nozzle/skimmer potentials yield neutral water loss.

**Scheme 1:**
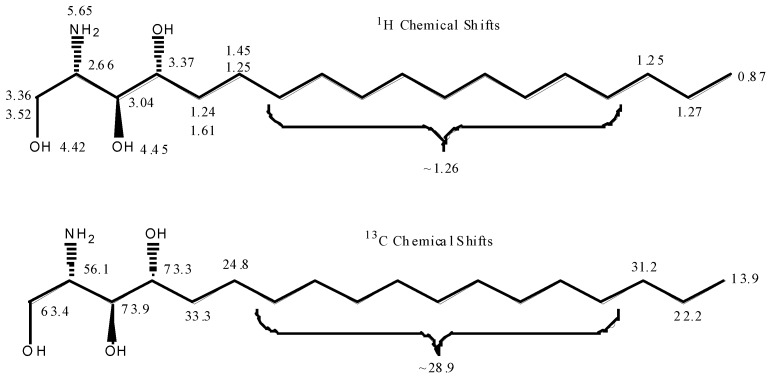
Phytosphingosine NMR chemical shifts.

Prior to monitoring the reaction of hexacosanoic acid and phytosphingosine, both starting materials were analyzed. Standard electrospray ionization of phytosphingosine, in which the analyte is dissolved in acetonitrile:water:acetic acid (50:50:0.1% *v*/*v*) and is sprayed through a central needle that is sheathed only in nitrogen gas for pneumatic assistance yielded a strong signal for [M + H]^+^ at *m*/*z* 318.2994 (theoretical 318.2989) with a signal to noise ratio (S/N) of 7000. Slightly raising the nozzle voltage provided in-source fragmentation with consecutive losses of water *m*/*z* 300.2846 and 282.2787, Figure 1. The structure and purity of phytosphingosine were also confirmed by NMR. Proton and carbon chemical shift assignments were made from the analysis of COSY, HMQC and HMBC spectra (using ~1.0 mg phytosphingosine dissolved in 0.65 mL DMSO-*d*_6_. as sample). The aliphatic region (0.8–1.8 ppm) of the proton spectrum integrates for 29 protons, 13 methylene groups, and a single methyl resonance are observed which are consistent with a straight chain (CH_2_)_13_CH_3_ alkyl side chain. The one-dimensional proton-decoupled carbon spectrum also confirms the presence of a straight chain alkyl side chain. The 0.7 ppm chemical shift dispersion observed for the central nine carbons of the alkyl chain indicate that no branching is present. The amino NH_2_ protons are visible as a very broad resonance at ~5.65 ppm. The ^1^H and ^13^C chemical shifts, proton coupling constants, ^1^H–^1^H COSY correlations and heteronuclear ^1^H–^13^C 2- and 3-bond correlations confirm the structure depicted in Scheme 1. NMR and mass spectral data were in agreement, indicating that the phytosphingosine starting material was pure with trace impurities limited to << 5%.

Due to the long aliphatic chain of the hexacosanoic acid, this starting material was not soluble in acetonitrile:water. The sample dissolved in tetrahydrofuran (THF) yielded [M − H + 2Na]^+^ at *m*/*z* 441.3699 (theoretical 441.3679) and the cluster species [2M − 2H + 3Na]^+^, *m*/*z* 859.7601 (data not shown for brevity).

### 2.2. Analysis of Reaction Products

Attempts were made to monitor the *N*-[3-(dimethylamino)propyl]-*N*′-ethylcarbodiimide hydrochloride/1H–1,2,3-benzotriazol-1-ol hydrate (EDC/HOBT) reaction and acid chloride reaction products by standard monospray ESI. Strong signals were observed for phytosphingosine; however, no signal was observed for the hexacosanoylceramide product expected at *m*/*z* 696, [M + H]^+^. These samples were only partially soluble in an acetonitrile:water mixture. In order to solubilize the hexacosanoic acid starting material and ceramide, approximately 1 mL of tetrahydrofuran (THF) was added to the 1 mL sample solution until dissolution was complete. The solubilized sample was sprayed by standard single needle design. Despite optimization of gas flow rates and nozzle/skimmer voltages, which were skewed towards higher nozzle voltages, only a minor signal was observed at *m*/*z* 696.6891 (theoretical 696.6864) with an *S*/*N* of 10 and a ^13^C isotope *S*/*N* of 4.0. This signal intensity is insufficient to unambiguously identify the product. Since no analytical methods were available to us to verify the product at the onset of the investigations, we sought alternate methods of ionization that we have at our disposal. The ionization schemes, attributes and pitfalls found for ceramides are discussed below.

### 2.3. Direct Laser Desorption Ionization Fourier Transform Ion Cyclotron Resonance Mass Spectrometry (FTICR MS) Analysis

**Figure 2:**
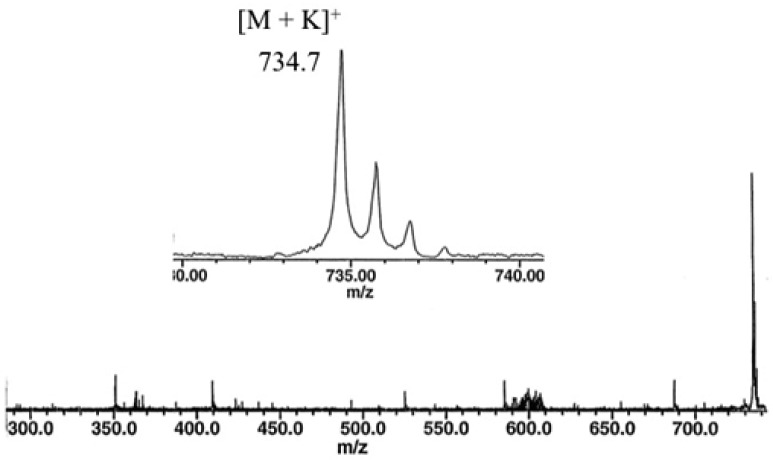
Direct laser desorption ionization of hexacosanoylceramide reaction mixture from EDC/HOBT coupling, reaction A. [M + K]^+^ is observed for the product, stable isotopic distribution is in agreement for the product.

**Figure 3:**
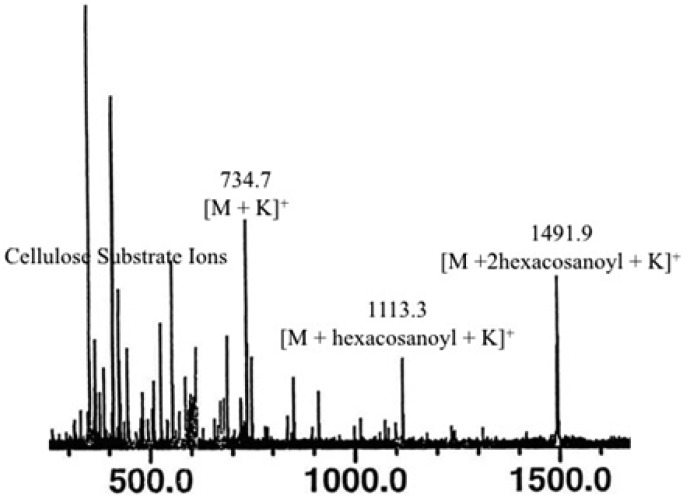
Direct laser desorption ionization (DLDI) mass spectrum of the hexacosanoylchloride coupling reaction B. [M + K]^+^, desired one covalent addition is observed for the product along with second and third covalent additions of hexacosanoyl to phytosphingosine.

Prior experience with highly apolar, water insoluble compounds, such as acrylic polymers, demonstrated that direct laser desorption ionization (DLDI) provides a rapid means of ionizing and characterizing such samples [35,36]. The technique is simply described as “dilute and shoot”, yielding representative data of polymer multicomponent samples. Matrix-assisted laser desorption ionization (MALDI) FTICR MS analysis of phospholipids has been successfully performed to yield high quality results. The identification of a proper matrix for the compounds is essential to the analysis [37]. DLDI does not suffer from matrix compatibility problems because the solubilized sample is placed directly on a cellulose substrate without a chemical matrix. Figure 2 shows that DLDI of the EDC/HOBT reaction yielded a significant signal at *m*/*z* 734.7 (theoretical 734.6), the targeted ceramide product, [M + K]^+^, with an *S*/*N* of ~200. The cellulose substrate which has strong absorbance at 10.6 µm (CO_2_ emission wavelength) functions as our matrix. The reaction mixture of hexacosanoyl chloride with phytosphingosine displayed additional signals at *m*/*z* 1113 and 1491, corresponding to the 2nd and 3rd covalent additions of hexacosanoyl to phytosphingosine (Figure 3). Accordingly, the EDC/HOBT coupling reaction was determined to be the best and was monitored further. Direct laser desorption mass spectrometry provided a rapid method to identify products from both reactions in a simple manner that takes less than twenty minutes to perform including sample preparation.

From the data, it is apparent that the solid cellulose substrate that holds the sample can randomly yield significant matrix ions, which could interfere with analysis. Second, the resolving power is much lower than expected for a 3T FTICR MS instrument due to laser desorption’s ionization mechanism and ion capture which yields ions that are not centered in the ICR cell, the homogeneous region of the magnetic field. Without quadrupolar axialization and collisional cooling to center the ions [38,39], the resolving power of DLDI is limited to ~3500 as the transient damps quickly to noise within 0.3 s. Mass measurement accuracy without internal reference ions were approximately 0.1 Da, dependent on mass measured. It is possible to use the matrix ions as an internal reference and achieve mass accuracies of ±0.01 Da; in this case, the observed mass was *m*/*z* 734.630 (theor. 734.642), data not shown for brevity. DLDI is also not amenable to online chromatography, which may be required due to sample solubility differences and the concentration ranges of the compounds under study. However, sample fractions could be collected and easily analyzed off-line.

### 2.4. Sheath Flow Electrospray Ionization

Sheath flow electrospray ionization was developed in the mid-1990s to improve ionization of intractable compounds by single needle ESI and to provide a simplified means to interface chromatographic methods such as gel permeation chromatography [34,40,41]. Prior attempts to improve electrospray ionization efficiency by adding sodium or potassium to the sample before separation showed that it can degrade the chromatography and compromise the column [42]. In sheath flow ESI, a triple needle design allows one to infuse the sample in an appropriate solvent or the chromatographic eluent through the center needle. The first sheath contains the ionization modifier solution such as sodium iodide or potassium iodide in methanol, all contained within the outer sheath of the nitrogen carrier gas. Separation of the ionizing agent and the analyte provides enhanced solubility of both components without the need to identify a solvent mixture that can solubilize both components in one vial. Ionization takes place in the Taylor cone through evaporation of solvents and mild ion/molecule association. Using this triple needle strategy, Ramjit et al. demonstrated improved analysis of apolar compounds [33].

**Figure 4:**
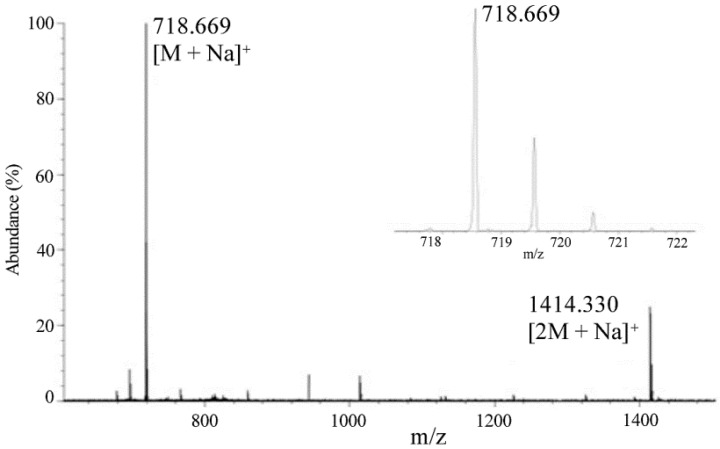
Sheath flow ESI mass spectrum of reaction A, mixture. [M + Na]^+^ is observed for the product. Note the improved resolving power compared to data in Figure 2.

Figure 4 shows the sheath flow ES FTICR mass spectrum of the EDC crude reaction mixture. Strong signals are observed for [M + Na]^+^ and [2M + Na]^+^. Improved resolving power (~10,000) and mass accuracy (2 mDa) is achieved by this technique with FTICR because the collected electrosprayed ions are now centered in the ICR cell.

### 2.5. Final Analyses

**Figure 5:**
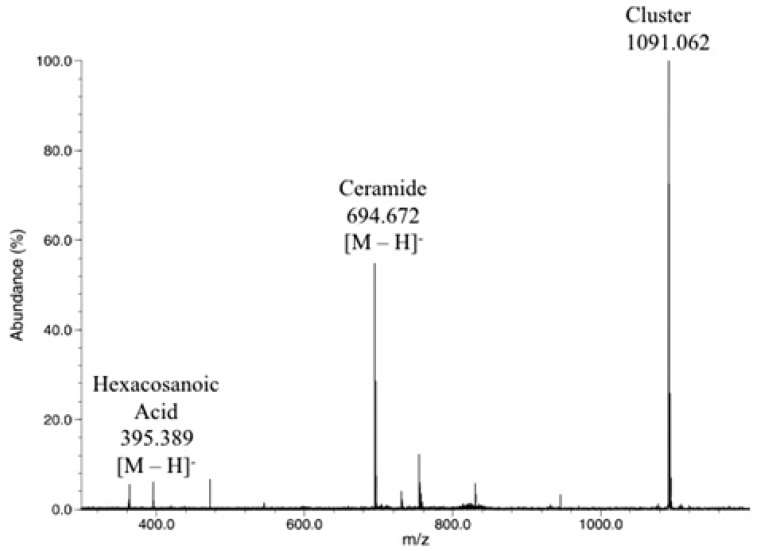
Negative ion sheath flow ESI mass spectrum of reaction A mixture. [M − H]^−^ is observed for residual hexacosanoic acid, hexacosanoylceramide product, and the cluster species [hexacosanoic acid + hexacosanoylceramide − H]^−^.

**Scheme 2:**
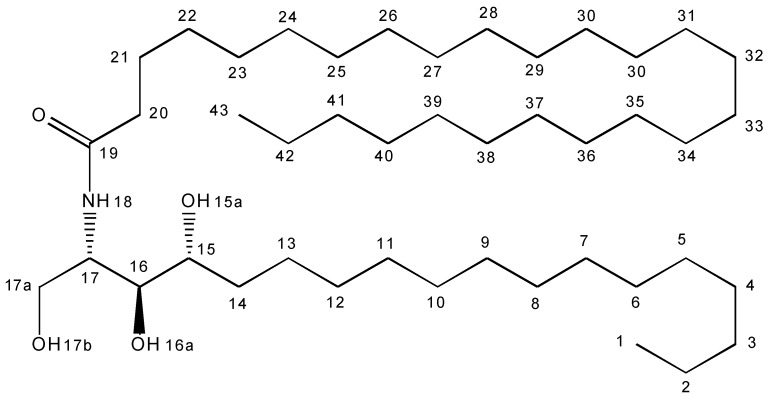
NMR hexacosanoylceramide carbon atom numbering scheme.

With improved mass spectral methods and a more thorough understanding of the sample complexity, the reaction chemistry was re-evaluated. In all cases, the combination of DLDI, Sheath Flow ESI-FTICR MS, and high field NMR analyses provided data consistent with the starting materials and desired product. NMR data acquired on THF solubilized samples generated complementary results. The ^1^H NMR spectrum recorded on the crude sample obtained from the EDC coupling revealed the presence of the two starting materials, hexacosanoic acid (~28%) and phytosphingosine (~4%) in addition to the desired product. Although proton integration was unreliable for quantifying the hexacosanoylceramide, the reaction product was easily identified. The amide proton is visible as a doublet at 6.93 ppm. There are two-bond HMBC correlations from the amide proton (6.93 ppm) and the methylene resonance (2.12 ppm) to the carbonyl carbon at 173.3 ppm, confirming the amide linkage. Three hydroxyl resonances are observed at ~4.20, ~4.11 and ~3.76 ppm. Specific assignments for each hydroxyl resonance were not possible due to the broad line widths resulting from chemical exchange broadening. Mass spectral data did not show the presence of starting material seen in Figure 4. Both sheath flow and monospray positive ion electrospray ionization of the hexacosanoic acid starting material yielded the expected [M − H + 2Na]^+^. In the presence of phytosphingosine or hexacosanoylceramide, only the molecular ions of these two compounds were observed, demonstrating preferential positive ionization over hexacosanoic acid. Negative ion sheath flow electrospray ionization yielded signals for both hexacosanoic acid and hexacosanoylceramide along with the cluster species (Figure 5). Molecular ionization efficiencies aside, data yields ~35% of residual hexacosanoic acid starting material, which is in close agreement with NMR results of the crude sample. Subsequently, the sample was recrystallized in THF (95% pure). The resolved proton chemical shifts were assigned from the results of COSY, HMQC and HMBC experiments (Scheme 2 and Table 1). Integration of the proton spectrum is consistent with the desired hexacosanoylceramide product (C_44_H_89_NO_4_). Mass spectral data of the purified sample was also in agreement.

## 3. Materials and Methods

### 3.1. Synthesis of N-[(1S,2S,3R)-2,3-Dihydroxy-1-(hydroxymethyl)heptadecyl] Hexacosanamide

#### 3.1.1. Samples via EDC/HOBT Coupling

**Scheme 3:**
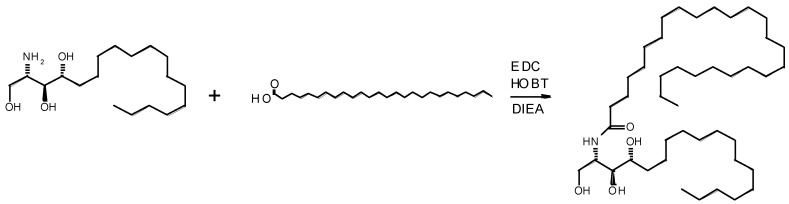
EDC/HOBT coupling reaction

The reaction is summarized in Scheme 3.

##### Starting Materials and Products

Phytosphingosine (C_18_H_39_NO_3_, 317.293 Da)
Hexacosanoic acid (C_26_H_52_O_2_, 396.396 Da)
Hexacosanoylceramide (C_44_H_89_NO_4_, 695.679 Da)

Hexacosanoic acid (0.125 g, 0.32 mmol), (2*S*,3*S*,4*R*)-2-aminooctadecane-1,3,4-triol (0.1 g, 0.32 mmol), *N*-[3-(dimethylamino)propyl]-*N*′-ethylcarbodiimide hydrochloride (EDC, 0.06 g, 0.32 mmol), 1H-1,2,3-benzotriazol-1-ol hydrate (HOBT, 0.048 g, 0.32 mmol), diisopropylethylamine (DIEA, 0.055 mL, 0.32 mmol) and 20 mL 1:1 DMF/THF were combined in a 50 mL round bottom flask fitted with a magnetic stirring bar and a nitrogen inlet. This mixture was stirred for 3 days, subsequently poured into 50 mL water and filtered. The resulting white solid was dried in vacuo to yield *N*-[(1*S*,2*S*,3*R*)-2,3-dihydroxy-1-(hydroxymethyl)heptadecyl]hexacosanamide (0.2 g, 0.29 mmol, 91%).

#### 3.1.2. Via the Acid Chloride

**Scheme 4:**
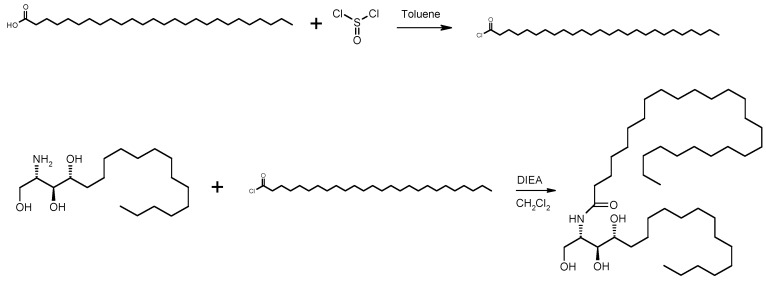
Hexacosanoylceramide synthesis via the acid chloride.

The reactions are summarized in Scheme 4. Hexacosanoic acid (0.125 g, 0.32 mmol) and 20 mL toluene were placed into a 50 mL round bottom flask fitted with a magnetic stirring bar and a nitrogen inlet. Thionyl chloride (0.046 mL, 0.63 mmol) was then added dropwise and the reaction heated to reflux for 3 days, cooled and the solvent removed in vacuo. The resulting crude reaction mixture was used in the next step without further purification.

(2*S*,3*S*,4*R*)-2-aminooctadecane-1,3,4-triol (0.1 g, 0.32 mmol), diisopropylethylamine (DIEA, 0.066 mL, 0.38 mmol) and 20 mL methylene chloride were placed in a 50 mL round bottom flask fitted with a nitrogen adapter and a magnetic stirring bar. The crude hexacosanoyl chloride was dissolved in 5 mL methylene chloride, added dropwise and the reaction stirred for 18 h. The mixture was poured into 50 mL water and the resulting white precipitate washed with water and dried in vacuo to yield *N*-[(1*S*,2*S*,3*R*)-2,3-dihydroxy-1-(hydroxymethyl)heptadecyl]hexacosanamide (0.12 g, 0.17 mmol, 54.7%).

### 3.2. Instrumentation

#### 3.2.1. 7T Bruker BioApex II™ FTICR MS

The 7T Bruker BioApex II™ FTICR mass spectrometer (Billerica, MA, USA) was equipped with a Bruker Apollo electrospray source. Experimental control and data processing were accomplished using Bruker XMass software (Version 6.10) running on Windows NT data stations.

#### 3.2.2. Finnigan FTICR MS NewStar

The 3T Finnigan Newstar FTICR mass spectrometer (ThermoFinnigan, San Jose, CA, USA) with the standard dual-trap configuration was equipped with a Tachisto CO_2_ (Needham, MA, USA) laser interfaced to the source trap and an Analytica ESI source (Branford, CT, USA) interfaced to the analyzer trap. Experimental control and data processing were accomplished using Odyssey software (Version 5.0) running on Sun Microsystems (Mountain View, CA, USA) computer stations.

#### 3.2.3. Nuclear Magnetic Resonance (NMR)

^1^H-NMR spectra were acquired on a Varian Unity Inova 600 MHz spectrometer (Varian Instruments, Palo Alto, CA, USA) equipped with a 5 mm triple resonance *Z*-axis pulsed field gradient probe. Proton-decoupled ^13^C spectra were acquired on a Varian Unity Inova 500 MHz spectrometer equipped with a Nalorac 5 mm triple nucleus ^13^C [^1^H/^19^F] probe (Nalorac Cryogens Corp., Martinez, CA, USA). All spectra were recorded at 25 °C.

### 3.3. MS Ionization Methods

#### 3.3.1. Standard Monospray Electrospray Ionization

Samples were dissolved in acetonitrile:water:glacial acetic acid (50:50:0.1% *v*/*v*) to provide ~50 µM concentrations. The Bruker Apollo ESI source operating conditions in the atmospheric pressure region of the source were: nitrogen concurrent gas 160 °C, 10 PSI, nitrogen countercurrent gas 160 °C, 30 flow rate (arbitrary units). Samples were delivered at 2–3 µL/min by use of a Cole Parmer 74,900 Series syringe pump to the single sheath needle assembly positioned 45° off-axis from the entrance capillary. The ESI needle was held at ground, while the endcap and capillary voltages were set at −3500 V and −4000 V, respectively. Nozzle or skimmer voltages in the 10^−6^ torr region of the source were held at 110 V, and 15.2 V, respectively. Ions were collected in the accumulation hexapole for approximately 0.5 s. The ion population was transferred to the Bruker ICR Infinity™ cell for analysis. In addition, 512 K data points were broadband detected at a Nyquist Frequency of 625 MHz (~*m*/*z* 200) yielding 0.42 s of acquisition time. Sixteen transients were co-added followed by Gaussian apodization and one zero fill prior to Fourier transformation. Accurate mass measurements were obtained by external calibration with polypropylene glycol of average mass 425 (Aldrich, Milwaukee, WI, USA).

#### 3.3.2. Direct Laser Desorption Ionization (DLDI)

The samples under study were dissolved in THF to yield approximately 10% *w*/*v* concentration. In addition, 100 µL of that solution and 100 µL of a saturated solution of potassium iodide used as the source of cations dissolved in THF were deposited on a cellulose substrate disk. To remove excess solvent, the sample disk was heated 15 min at 110 °C. The sample disk was positioned 3 mm from the source trap plate of the ICR cell via an automatic insertion probe. The Tachisto CO_2_ laser beam (λ = 10.6 µm, power 10^6^–10^8^ watts/cm^2^, pulse width = 40–80 ns) was fired at the sample disk producing a spot size of approximately 1 mm^2^. The potassiated molecular ions were collected and allowed to equilibrate for 2.5 s in the cell. All ions below 300 *m*/*z*, primarily due to KI, were ejected by a radio-frequency excitation pulse. After a 30 ms delay to allow ions to equilibrate in the ICR cell, the remaining ions were rf-excited and detected. Thirty-two K data points were acquired at a Nyquist Frequency of 347 kHz, and one zero fill was added before Fourier transformation. The transients were not apodized. Only mono-potassiated signals were observed. For a more detailed description of the DLDI-FTICR MS experiments, consult Simonsick and Ross [35] and the CO_2_ laser references within.

#### 3.3.3. Sheath Flow Electrospray Ionization

The samples under investigation were dissolved in THF to a concentration of approximately 50 µg/mL. Sodium iodide was added to a methanol solution to yield a concentration of 15 µg/mL (50 µM). The ESI needle was held at ground, while the endcap, capillary, and cylinder voltages were set at −3200, −4000, and −2150 V, respectively. Nozzle and skimmer voltages in the 10^−6^ torr region of the source were held at 220 V, and 10 V, respectively. The Analytica ESI source was fitted with a triple sheath needle assembly and interfaced to the Finnigan FTMS Newstar system. The samples were sprayed through the inner needle at 15 µL/min, the sodium iodide solution in the middle sheath at 30 µL/min, and the nitrogen concurrent/sheath and countercurrent gases were held at 60 PSI. Each acquisition contained 128 K data points with a lower limit of *m*/*z* 200. Twenty transients were co-added and one zero fill was performed prior to Fourier transformation. Negative ion sheath flow ESI used pure methanol as the sheath liquid with endcap, capillary and cylinder, nozzle/skimmer voltages of +2800, +3900, +2050, −220, and −10 V, respectively.

### 3.4. NMR Analysis

Samples for NMR measurements contained approximately 1 mg of phytosphingosine dissolved in DMSO-*d*_6_ (Isotec 99.96 atom % D) or 1.5 mg of hexacosanoylceramide dissolved in THF-*d*_8_ (Isotec 100.0 atom % D) in a final sample volume of 0.650 mL. ^1^H and ^13^C chemical shifts were referenced to tetramethylsilane (TMS) at 0.00 ppm. One-dimensional carbon spectra were acquired with 72 K data points, a sweep width of 28 kHz and 25,000 transients using a combined recycle delay and acquisition time of 2.3 s. Broadband proton decoupling was achieved by the application of a globally optimized alternating phase rectangular composite pulse (GARP) modulation scheme [43] during the acquisition period and recycle delay.

Proton chemical shift assignments were obtained with pulsed-field gradient correlated spectroscopy (PFG-COSY) [44]. PFG-COSY spectra were acquired with 1 K complex points in t_2_ and 1024 points in t_1_ consisting of 1–8 transients per increment. The data was multiplied by a sine-bell apodization function in both dimensions and zero-filled to 4 K by 2 K points prior to Fourier transformation.

Carbon chemical shift assignments were made with proton-detected ^1^H–^13^C heteronuclear multiple quantum coherence spectroscopy (HMQC) [45] and pulsed field gradient heteronuclear multiple bond correlation spectroscopy (PFG-HMBC) [46]. Broadband carbon decoupling in the HMQC spectra was accomplished with a GARP 1 [43] composite pulse decoupling scheme. HMQC spectra were recorded with 1 K complex points in t_2_ and 256 increments in t_1_ with 4–8 transients per increment. The fixed delays were optimized for a ^1^H–^13^C 1-bond coupling constant of 140.0 Hz. The data were zero-filled to 2 K complex points in t_2_ and 1 K complex points in t_1_ and were multiplied by a Gaussian function in both dimensions prior to Fourier transformation. PFG-HMBC spectra were acquired in the absolute value mode with 1 K complex points in t_2_ and 1024 increments in t_1_ with 8–48 transients per increment. The heteronuclear multiple bond fixed delay was set to 65.0 ms to optimize observation of 2- and 3-bond heteronuclear correlations. All two-dimensional experiments were acquired in the absolute value mode with the exception of the HMQC experiment which was acquired in the hypercomplex mode for phase-sensitive presentation.

## 4. Conclusions

The water soluble phytosphingosine starting material ionizes well by use of standard monospray positive ion electrospray conditions. The non-water soluble starting materials hexacosanoic acid and ceramide reaction products were observed by the use of direct laser desorption and sheath flow electrospray ionization. To visualize all the components in one spectrum, negative ion sheath flow ESI provided the best results and could be interfaced to chromatography if deemed necessary. No single ionization method is a panacea for characterizing all samples, particularly compounds with such different physicochemical properties as discussed here. NMR and mass spectral analysis are complementary. The combination of FTICR MS’s high mass accuracy and resolving power with direct laser desorption ionization, sheath flow electrospray ionization, and high field NMR studies provide an efficient methodology for structural characterization of these important biological lipids.

## Figures and Tables

**Table 1:** Carbon and proton chemical shifts for hexacosanoylceramide in THF-*d*_8_^1^.

Position	^13^C (ppm)	^1^H (ppm)
1	14.5	0.89
2	23.7	1.32
3	32.9	1.3
4–12	~30.7	~1.31
13	26.7	1.32, 1.53
14	34.3	1.33, 1.71
15	73.2	3.44
15a		3.77
16	77.2	3.41
16a		4.21
17	53.9	4.01
17a	62.5	3.61, 3.71
17b		4.12
18		6.93
19	173.3	
20	36.7	2.12
21	26.6	1.59
22–40	~30.3	~1.31
41	32.9	1.3
42	23.7	1.32
43	14.5	0.89

^1^ Carbons 1–3 and 40–43 were assigned by comparison of the HMBC spectra of the two starting materials, phytosphingosine and hexacosanoic acid, and the final product hexacosanoylceramide.

## Abbreviations

DLDI	Direct laser desorption ionization
ESI	Electrospray ionization
FTICR MS	Fourier transform ion cyclotron resonance mass spectrometry
HPLC	High performance liquid chromatography
TLC	Thin layer chromatography
NMR	Nuclear magnetic resonance
EDC	*N*-[3-(dimethylamino)propyl]-*N*′-ethylcarbodiimide hydrochloride
HOBT	1H–1,2,3-benzotriazol-1-ol hydrate
MALDI	Matrix-assisted laser desorption ionization
PFG-HMBC	Pulsed field gradient heteronuclear multiple bond correlation spectroscopy
HMQC	2D heteronuclear multiple quantum coherence spectroscopy
HMBC	2D heteronuclear multiple bond correlation spectroscopy
COSY	2D homonuclear correlation spectroscopy
GARP	Globally optimized alternating phase rectangular pulse
